# Unusual developmental morphology and anatomy of vegetative organs in *Utricularia dichotoma*—leaf, shoot and root dynamics

**DOI:** 10.1007/s00709-019-01443-6

**Published:** 2019-10-28

**Authors:** Markus S. Reut, Bartosz J. Płachno

**Affiliations:** Department of Plant Cytology and Embryology, Institute of Botany, Faculty of Biology, Jagiellonian University, Kraków, 9 Gronostajowa St, 30-387 Cracow, Poland

**Keywords:** *Utricularia*, *Utricularia dichotoma*, *Polypompholyx*, *Pleiochasia*, Bladderworts, Dynamic morphology

## Abstract

The terrestrial carnivorous species *Utricularia dichotoma* is known for a great phenotypic plasticity and unusual vegetative organs. Our investigation on 22 sources/populations revealed that after initiation of a leaf and two bladders on a stolon, a bud was formed in the proximal axil of the leaf, developing into a rosette with up to seven organs. The first two primordia of the bud grew into almost every possible combination of organs, but often into two anchor stolons. The patterns were generally not population specific. The interchangeability of organs increased with increasing rank in the succession of organs on stolon nodes. A high potential of switching developmental programs may be successful in a fluctuating environment. In this respect, we were able to show that bladders developed from anchor stolons experimentally when raising the water table. Anatomical structures were simple, lacunate and largely homogenous throughout all organs. They showed similarities with many hydrophytes, reflecting the plant’s adaptation to (temporarily) submerged conditions. The principal component analysis was used in the context of dynamic morphology to illustrate correlations between organ types in the morphospace of *U. dichotoma*, revealing an organ specific patchwork of developmental processes for typical leaves and shoots, and less pronounced for a typical root. The concept and methods we applied may prove beneficial for future studies on the evolution of Lentibulariaceae, and on developmental morphology and genetics of unusual structures in plants.

## Introduction

Due to the very special plant architecture, the genus *Utricularia* L. (Lentibulariaceae) has been and still is a popular subject to study developmental morphology (e.g. Darwin 1875; Goebel 1891, 1898, 1905; Lloyd 1942; Chormanski and Richards 2012; Rutishauser 2016), embryology (e.g. Kamieński 1877; Merz 1897; Lang 1901; Kondo et al. 1978; Płachno and Świątek 2010, 2011; Płachno 2011), histology/ultrastructure (e.g. Fineran 1980; Płachno et al. 2017a, 2017b), and molecular phylogeny/genetics (e.g. Jobson and Albert 2002; Carretero-Paulet et al. 2015a, 2015b; Ibarra-Laclette et al. 2011, 2013; Silva et al. 2018).

As a fascinating ambassador of the ‘carnivorous syndrome’ (Juniper et al. 1989), *Utricularia* had evolved small bladders with an extremely fast suction mechanism to trap organisms in the water or wet soil (e.g. Poppinga et al. 2015). Depending on the life form and species, *Utricularia* vegetatively develops various additional organ types such as (foliage) leaves, runner stolons, water shoots, floats, rhizoids (in some species called ‘anchor stolons’), air shoots, turions and tubers (Taylor 1989; Rutishauser 1993; Rutishauser 2016). Mature plants of the sister genus *Genlisea* A.St.-Hil. have a less complex vegetative body with rosulate foliage leaves and leaf-like invers Y-shaped ‘rhizophylls’ showing homologies with the *Utricularia* bladder and functioning like an eel-trap in wet substrate (Lloyd 1942; Reut 1993; Fleischmann 2018). A stoloniferous habit is only known from *G. repens* which produces an elongated rhizome (Reut 1993; Fleischmann 2018). In contrast to *Genlisea* and *Utricularia*, the third genus within the family, *Pinguicula* L., traps the prey on its generally rosulate and sticky foliage leaves above ground, and grows roots into the soil (Fleischmann and Roccia 2018). However, some species of *Pinguicula* have reduced roots lacking a root cap and/or root branches (e.g. *P. moranensis*: Rutishauser and Isler 2001; Rutishauser 2016).

Whilst the structural bauplan of most angiosperms follows the classical concept with the basic organ classes ‘shoot’ (‘leaf’ and ‘shoot axis’/‘stem’) and ‘root’, the bladderworts are ‘morphological misfits’, since their vegetative organs seem to represent mixtures, fuzzy combinations or continuums of the basic categories, i.e. strict ‘either-or’ assignments to the basic organ classes fail (e.g. Rutishauser 2016). For instance, *Utricularia* lacks ‘typical’ roots, e.g. with root caps, root hairs and endogenous branching (Lloyd 1942; Taylor 1989; Adlassnig et al. 2005), but some stolon types still resemble roots in several aspects (Rutishauser 2016). Therefore, in view of continuum or fuzzy morphology, developmental programs for roots were not completely lost but amalgamated with processes for shoots, at least in some species (Rutishauser 2016). Within the genus *Utricularia*, terrestrial species of subgenus *Bivalvaria* section *Calpidisca*, epiphytic species of subgenus *Utricularia* sections *Orchidioides*, *Psyllosperma*, and *Iperua*, and aquatic species of subgenus *Utricularia* sections *Utricularia* and *Vesiculina* have been subject to developmental morphological studies and discussions in the context of fuzzy, continuum, or dynamic morphology (Brugger and Rutishauser 1989; Rutishauser and Sattler 1989; Sattler and Rutishauser 1990; Jeune and Sattler 1992; Sattler and Jeune 1992; Rutishauser 1993; Rutishauser 1999; Rutishauser and Isler 2001; Jeune et al. 2006; Kirchoff et al. 2008; Rutishauser 2016). Rather than being purely descriptive, Sattler and Rutishauser (1990) were the first to use diagrams to illustrate differences of combinations of morphogenetic processes (growth modalities/parameters) between vegetative organs of the two aquatic species *U. foliosa* and *U. australis*. Bracts, water-shoots, dissected leaves and air-shoots of *U. foliosa* were part of the early multivariate analyses on developmental processes to quantitatively reveal the diversity of plant forms in the sense of dynamic morphology (Jeune and Sattler 1992; Sattler and Jeune 1992; Jeune et al. 2006). The studies differ in some of the selected parameters and in the scope of organs covered (e.g. Jeune et al. 2006 excluded roots), but they demonstrate that typical roots, stems, shoots, leaves (and trichomes) build clusters and atypical structures are intermediates between typical organ categories. The current study focuses on the terrestrial *U. dichotoma* Labill. of subgenus *Polypompholyx* Lehm. section *Pleiochasia*, since (a) *Polypompholyx* is considered to have the closest morphological affinity to the *Genlisea*-*Utricularia* ancestor (cf. Goebel 1898, 1905; Merl 1915; Taylor 1989; Jobson et al. 2018), (b) *Utricularia dichotoma* has a great phenotypic plasticity and the widest distribution amongst species of subgenus *Polypompholyx* (Reut and Fineran 2000; Jobson et al. 2017), and (c) compared to rosette plants of other sections in *Polypompholyx* and to *U. foliosa*, *U. dichotoma* grows stolons with several types of organs (Reut and Fineran 2000) of which the morphological significance has not yet been determined in the context of fuzzy or dynamic morphology.

*Utricularia* subg. *Polypompholyx* was treated as separate genus for more than a century, mainly due to mature plants having four calyx lobes versus two lobes in subgenus *Utricularia*, maybe representing an intermediate form of genus *Genlisea* and *U.* subg. *Utricularia* (Taylor 1989). Recent molecular data supported the placement of *Polypompholyx* as subgenus within *Utricularia* (Müller et al. 2006; Jobson et al. 2017). Jobson et al. (2017) confirmed *Polypompholyx* (Lehm.) P. Taylor and *Tridentaria* P. Taylor to be sister sections within the subgenus. Moreover, section *Pleiochasia* Kamieński sensu Taylor (1989) was identified as sister to the *Polypompholyx-Tridentaria* clade (Jobson et al. 2017; Silva et al. 2018), and *Pleiochasia* was divided into the two sections *Pleiochasia* Kamieński and *Lasiocaules* R.W.Jobson and Baleeiro (Jobson et al. 2017).

*Utricularia dichotoma* sensu lato (including *U. monanthos* and *U. novae-zelandiae*) of section *Pleiochasia* occurs on the Australian mainland (South West Australia, Victoria, New South Wales and Queensland) and extends to Tasmania, New Zealand and (probably still) New Caledonia (Taylor 1989; Jobson et al. 2017). The species preferably occupies nutrient poor wetlands in open vegetation such as lakesides, river shores, heathland, swamps, pools in mountain bogs, or roadside ditches (Taylor 1989; Reut and Fineran 2000). Depending on the habitat, the plants grow in substrate, which is permanently saturated, temporarily submerged, or just humid or occasionally dry (Reut and Fineran 2000). The evolutionary shift from the terrestrial to the aquatic habit may have led to a higher phenotypic plasticity and organ complexity in the genus (cf. Jobson et al. 2018), in which the stoloniferous *U. dichotoma* seems to adopt an intermediate role towards more ‘advanced’ terrestrial *Utricularia* species (Merl 1915; Taylor 1989). Recognising that *U. dichotoma* shows a great morphological diversity, Taylor (1989) revised the taxonomy of this species and pointed out uncertainties with respect to the distinction of *U. dichotoma*, *U. monanthos* Hook.f. and *U. novae-zelandiae* Hook.f., due to limited material at hand. Other botanists had difficulties in differentiating between *U. monanthos* and *U. novae-zelandiae* (Johnson and Brooke 1989; Wardle 1991; Reut 1995; Webb and Sykes 1997) and between *U. monanthos* and *U. dichotoma* (Cochrane et al. 1968). Reut and Fineran (1999) investigated taxonomical relationships of populations of *U. dichotoma*, *U. monanthos* and *U. novae-zelandiae* from New Zealand and Australia (Tasmania, Victoria and New South Wales) and concluded that the three species should be placed under *U. dichotoma*, since a clear distinction of these taxa was not possible based upon previously acknowledged traits (e.g. number of inflorescences per plant, plant size, flower dimensions and colour, and leaf characters). This proposal was followed by Salmon (2001) and reflected by molecular data of plastid DNA by Reut and Jobson (2010) and Jobson et al. (2017). Therefore, *U. monanthos* and *U. novae-zelandiae* are treated as synonyms of *U. dichotoma* in the current study.

*Utricularia* subg. *Polypompholyx* shows several types of vegetative organs, of which the terminology is provided in ‘Materials and Methods’. Members of sections *Polypompholyx* and *Tridentaria* and some species of section *Pleiochasia* grow as rosette plants with foliage leaves, anchor stolons (rhizoids), bladders and inflorescences deriving from a short stem (Taylor 1989; Jobson et al. 2018). However, species of section *Lasiocaules* and most species of section *Pleiochasia*, including *U. dichotoma*, additionally produce runner stolons which may carry leaves, several types of branched and unbranched stolons, bladders and occasionally inflorescences (Merl 1915; Lloyd 1942; Taylor 1989; Reut and Fineran 2000; Jobson et al. 2018). In determined position on a stolon of terrestrial and aquatic utricularias, types and numbers of organs have been found to vary to a certain degree (e.g. Brugger and Rutishauser 1989; Rutishauser 2016). This variability was confirmed by Reut and Fineran (2000) on plants from *U. dichotoma* and thought to be correlated with the micro-habitat.

The great phenotypic plasticity and adaptability of *Utricularia* are associated with a high diversity of about 240 species, as it is the largest genus of carnivorous plants with an almost worldwide occurrence in generally nutrient-poor, wet to aquatic and open habitats (Reut and Fineran 2000; Poppinga et al. 2015; Rutishauser 2016; Jobson et al. 2018). Reut and Fineran (2000) outlined the developmental morphology and types of organs on stolons of *U. dichotoma* from eight sites in New Zealand and from one site in Tasmania. The early development of one foliage leaf and two flanking bladders on the stolon tip was described by Merl (1915) and confirmed by Reut and Fineran (2000). Lloyd (1942) reported another pattern of organ development in *U. dichotoma* and allied species, but his illustrations of *U. monanthos* do not fully conform with his description, for which he made no exact reference to the material studied. In addition, Lloyd (1942) observed that bladders occasionally arise on the upper side of internodes of branched stolons of *U. dichotoma*. This was described by Taylor (1989) for several species of section *Pleiochasia* sensu Taylor (1989), but it was not reported by Reut and Fineran (2000) on plants of *U. dichotoma*. Instead, Reut and Fineran (2000) found ‘simple stolons’ in *U. dichotoma* which carry a few bladders along their axis, seemingly representing an intermediate, unbranched form of anchor and runner stolons. Lloyd (1942) mentioned that an inflorescence may arise from the leaf axil on the stolon node, and in his drawings of *U. monanthos*, he also showed the initiation of the peduncle from the primary shoot of the seedling. In the current study, we examined the morphology of vegetative organs of *U. dichotoma* by including plants from more sites of origin than previously investigated (cf. Merl 1915; Lloyd 1942; Reut and Fineran 2000) and by using scanning electron microscopy to gain insight into leaf-, shoot- and root-like structures and their developmental patterns.

The various vegetative organs developed by *U. dichotoma* give rise to questions with respect to the classical root-shoot model, e.g., is the bladder (the trap) homologous to a whole leaf? Does an anchor stolon share morphological and developmental characters to a shoot *or* a root? Is the runner stolon equivalent to a shoot or a (compound) leaf? Is the simple stolon, bearing bladders, a root, a leaf, or a shoot? However, since the vegetative organs rather represent a mixture of categories, the question ‘how far is it this or that?’ shall be answered for all vegetative organ types of *U. dichotoma* in this paper by applying the mathematical approach of the principal component analysis (PCA) in the concept of dynamic/process morphology following, e.g. Jeune and Sattler (1992) and Jeune et al. (2006). This method enables a visualization of correlations (and distances) between plant structures and groups of organs in the morphospace based upon developmental processes (Kirchoff et al. 2008).

Histological studies covering *U. dichotoma* are scarce. Lloyd (1942) and Merl (1915) showed that the structures of leaves, stolons and petioles are simple. Recently, Płachno et al. (2014, 2017a) described trap walls and vascular tissue of *U. dichotoma* (including two clones from New South Wales, Australia). In the investigated species of sugenus *Polypompholyx*, the bladder showed one vascular strand with well-developed xylem elements, running along the dorsal bladder side (Płachno et al. 2017a). In our study, we investigated anatomical structures of stolons, leaves and bladder stalks of *U. dichotoma* to contribute to the overall understanding of the nature of these vegetative organs, and all data was analysed based on the PCA approach.

## Materials and methods

### Plant material

To prepare the material of *Utricularia dichotoma *for examinations, it was cleaned from soil particles, algae and other plants by careful rinsing with water (fresh material only) and manual separation under a stereo microscope.

Plant material was visually inspected with a stereo microscope to identify and draw the developmental patterns of vegetative organs, and to choose samples for further investigations by light microscopy (LM) and scanning electron microscopy (SEM). All material in 70% ethanol (cf. Table 1) was used for morphological studies.

**Table 1:** Plant material of *U. dichotoma* from 24 sources used for investigations, with information on sites of origin and initial preparation/preservation

Voucher	AB	Source, locality
B. Salmon 116	Oh	Lake Ohia, NZ NI^d^ (SEM)
B.A. Fineran and M.S. Reut 95501	Ko	Kopouatai Peat Dome, NZ NI^a, e^ (SEM)
P.J. de Lange 869	Wh	Te Kauwhata, Whangamarino Wetlands, NZ NI^d, f^
B. Salmon 374	Bl	Blyth Swamp, Mt Ruapehu, NZ NI^d^ (SEM)
M.S. Reut and B. Salmon 03951	Bo	Bog Inn, Pureora Forest, NZ NI^d^ (SEM)
M.S. Reut and B. Salmon 03952	Up	Upper Link Track, Pureora Forest, NZ NI^d^
B.A. Fineran and M.S. Reut 95500	To	Tawhai Falls, Tongariro National Park, NZ NI^a, e^
B.A. Fineran and M.S. Reut 95355	De	Denniston, NZ SI^a, e^
B.A. Fineran and M.S. Reut 95354	Ok	Okarito, NZ SI^a, e, g^
B.A. Fineran and M.S. Reut 95356	La	Lake Pearson, NZ SI^a, e^
B.A. Fineran and M.S. Reut 95353	Gr	The Groynes, NZ SI^a, e^
B.A. Fineran and M.S. Reut 95844	Te	Te Anau, NZ SI^a, e^
M.S. Reut and J. Flisek 271		Stewart Island, Scott Burn, NZ^b, g^
B.J. Płachno and K. Pasek 333		From Botanical Garden of Jagiellonian University, Cracow, Poland. Ex origin, NZ^b, c, h^ (SEM)
M.S. Reut 101 (water saturated soil)	Ns	From M.S. Reut private cultivation, Switzerland. Ex B.J. Płachno and K. Pasek 333. Ex origin, NZ^b^
M.S. Reut 102 (wet soil)	Nw	From M.S. Reut private cultivation, Switzerland. Ex B.J. Płachno and K. Pasek 333. Ex origin, NZ^b^
M.S. Reut and J. Flisek 272	Qu	Queenstown, Tas^b, g^ (SEM)
A. Rozefelds and M.S. Reut 95734	St	Strathgordon, Tas^a, f^ (SEM)
B. Salmon 390 Var. ‘Tina alba’		The Grampians, Vic^d^ (SEM)
B.J. Płachno and L. Adamec 512 ‘Robust clone’	Ne	From Lubomir Adamec collection, Cech Republic. Ex origin, Newcastle, NSW^c, h^ (SEM)
B.J. Płachno and M.S. Reut 617 ‘Smaller clone’	Ka	From Botanical Garden of Jagiellonian University, Cracow, Poland. Ex origin, Katoomba, NSW^b, h^
B.J. Płachno and K. Pasek 334	Fa	From Botanical Garden of Jagiellonian University, Cracow, Poland. Ex origin, Falls Creek, Vic^b, c^ (SEM)
B.J. Płachno and K. Pasek 335	An	From Botanical Garden of Jagiellonian University, Cracow, Poland. Ex origin, Anglesea, Vic^b, c^ (SEM)
B.J. Płachno and K. Pasek 336 Var. ‘Chestnut’, big flower.	Ch	From Botanical Garden of Jagiellonian University, Cracow, Poland. Ex origin, Vic^b, c^ (SEM)
M.S. Reut and B. Matter 202	Es	From Botanical Garden, Basel, Switzerland. Ex origin, Esperance, WA^b^

*AB* abbreviation of source name, used in Fig. 5; *NZ* New Zealand; *NI* North Island; *SI* South Island; *AUT* Australia; *Tas* Tasmania; *Vic* Victoria; *NSW* New South Wales; *WA* Western Australia, *SEM* material used for scanning electron microscopy

^a^Fresh material transferred in 70% ethanol on site

^b^Fresh material received from collection/cultivation and transferred in 70% ethanol

^c^Fresh material received from collection/cultivation and transferred in 2.5% glutaraldehyde, 2.5% formaldehyde, and used for anatomical studies (light microscopy)

^d^Material from spirit collection (70% ethanol)

^e^cf. Reut and Fineran (1999)

^f^cf. Reut and Fineran (2000)

^g^cf. Reut and Jobson (2010)

^h^cf. Płachno et al. (2014, 2017a)

For LM, the plant material (cf. Table 1) was fixed in a mixture of 2.5% glutaraldehyde with 2.5% formaldehyde in a 0.05 M cacodylate buffer (Sigma; pH 7.2) overnight or for several days, washed three times in a 0.1 M sodium cacodylate buffer and post-fixed in a 1% osmium tetroxide solution at room temperature for 1.5 h. The material was dehydrated in a graded ethanol series to 95%, infiltrated and embedded using an epoxy embedding medium kit (Fluka). Following polymerisation at 60 °C, sections were cut using a Leica ultracut UCT ultramicrotome. Semi-thin sections (0.9–1.0 μm thick) were prepared for LM and stained for general histology using aqueous methylene blue/azure II (MB/AII) for 1–2 min (Humphrey and Pittman 1974) and examined with an Olympus BX60 light microscope. Some material for semi-thin sections was fixed in ethanol/acetic acid (3:1, *v*/*v*).

For SEM, the plant material (cf. Table 1) was fixed (as above or in ethanol) and later dehydrated and subjected to critical-point drying using liquid CO_2_. They were then sputter-coated with gold and examined at an accelerating voltage of 20 kV using a Hitachi S-4700 scanning electron microscope (Hitachi, Tokyo, Japan), which is housed in the Institute of Geological Sciences, Jagiellonian University, in Kraków. Material from Strathgordon, Tasmania (A. Rozefelds and M.S. Reut 95734), was critical-point dried at the Department of Systematic and Evolutionary Botany, University of Zurich, Switzerland, in 1997. Pictures of stolons were taken with a Phillips scanning electron microscope at the University of Neuchâtel, Switzerland, in the same year.

### Cultivation experiment

A clone (M.S. Reut 102) from the B.J. Płachno and K. Pasek 333 cultivation was transferred to an in-door aquarium at the home of M.S. Reut in Switzerland in April 2018, where it was propagated at room temperature in a pot of a peat-sand 2:1 mixture with a water table (distilled water-tap water 9:1) about 4 cm below the soil surface. This substrate was constantly wet but not saturated. The light source was a Hagen Flora-Glo 2800K bulb with photosynthetic spectrum, 420 lm and 65 lx. After 4 weeks, one part (M.S. Reut 101) was moved to a lower pot with the soil surface at water level, propagated in this water-saturated to slightly submerged condition for 8 weeks, and then prepared in 70% ethanol. Another 4 weeks later, a part of the original clone M.S. Reut 102 was also transferred into 70% ethanol. Clones M.S. Reut 101 and M.S. Reut 102 underwent visual examination with a Kern OZL 464 stereo/trinocular microscope.

### Terminology

The terminology of vegetative organs of *Utricularia dichotoma *follows Reut and Fineran (2000) (Table 2). The primary shoot arising from the embryo, or the shoot from where an organ originates, is regarded as reference axis. Therefore, the terms ‘proximal’ and ‘distal’ are used in relation to this axis of origin. Radford et al. (1974), Wagenitz (1996), and Reut and Fineran (2000) define ‘dorsal’ as the lower or abaxial side (and ‘ventral’ as the upper or adaxial side) of an organ (especially leaf). However, in botanical literature, ‘dorsal’ is often used contradictory (cf. Gleissberg et al. 1999). Therefore, we use ‘dorsal’ and ‘ventral’ only in the term ‘dorsiventral’ (the ab-adaxial axis) indicating that the upper and lower sectors (of a stolon) are differently structured. The terms ‘up’ and ‘down’ refer to as geotropically negative and positive, respectively. ‘Nodes’ are usually defined as generative zones on runner stolons, from where various organs may arise. If a secondary runner stolon develops from a runner stolon, the parent stolon is called a ‘branched stolon.’ In our paper, ‘branching’ is used whenever an axis gives rise to a daughter structure.

### Data analysis

In order to identify and measure correlations between vegetative organs of *Utricularia dichotoma* and typical roots, shoots and leaves, and their respective developmental processes (syn. modalities, parameters, or variables; see Table 3), the principal component analysis (PCA) was applied, largely following Jeune and Sattler (1992). In contrast to the PCA of Jeune et al. (2006), who centred the data set on the ‘theoretical shoot’ (focussing on the partial-shoot concept of all organs) and excluded roots, we included all basic organ categories (shoot, leaf and root) without centring any of them. Whilst Sattler and Jeune (1992) and Jeune et al. (2006) selected representative species for typical structures, we followed Jeune and Sattler (1992) and used a larger variety of a typical (although theoretical) root, shoot and leaf in angiosperms with a broader range of possible combinations of modalities per organ type (see Table 4), leading to larger areas of the organ types in the morphospace. The PCA data set (Table 4) was complemented with the reduced root of *Pinguicula moranensis* (to assess correlations with the typical root and *Utricularia* stolons), and all vegetative organs of *U. dichotoma* including the inflorescence axis (see Table 4). Due to different positionings (Table 3, modality 1) of certain organs on stolons (cf. Reut and Fineran 2000), we distinguished between subtending (axillant) organs (e.g. the first leaf on a stolon node) and secondary (axillary) organs (e.g. a leaf developing from the base of the first leaf on the stolon node) in the data set (Table 4).

**Table 2:** Terms used by several botanists for vegetative organs relevant for *U. dichotoma*

Reut and Fineran (2000)	Goebel (1891, 1898, 1905)	Lloyd (1942)	Taylor (1989)
Foliage leaf, leaf	Foliage-leaf (‘Laubblatt’)	Leaf	Leaf
Bladder, trap	Tube (‘Schlauch’), tubular leaf (‘Schlauchblatt’) ending in a bladder (‘Blase’)	Trap	Trap
Anchor stolon	Leaf-root (‘Blattwurzel’)^a^, rhizoid (‘Rhizoid’)	Anchoring stolon, branch stolon (when branching from a runner stolon)	Rhizoid
Runner stolon	Runner (‘Ausläufer’)	Runner stolon, branch stolon (when branching from a runner stolon)	(Branched) stolon
Simple stolon	Stolon in transition from leaf-root to runner, bladder bearing stolons (‘Blasen tragende Blätter’)	Not described for *U. dichotoma*	Not described for *U. dichotoma*

^a^Merl (1915) also uses the term root-leaf (‘Wurzelblatt’)

**Table 3:** Modalities (developmental processes) and their values used in the PCA

	Modality	Value
(1)	Positioning^a^	Other (− 1.0), main axis (− 0.5), axillary (+ 0.5), axillant (+ 1.0)
(2)	Growth period^b^	Indeterminate (− 1.0), determinate (+ 1.0)
(3)	Orientation^b^	Inward / no cap (− 1.0), inward and outward / cap (1.0)
(4)	Geotropism (gravitropism)^b^	Negative (− 1.0), zero (0.0), positive (1.0)
(5)	Developmental symmetry^a^	Variable/changing (− 0.5), stable/non changing (+ 0.5)
(6)	Final symmetry^a^	Radial (− 0.5), dorsiventral (+ 0.5)
(7)	Growth distribution^a^	Mixed = acropetal and basipetal (− 1.0), acropetal (− 0.5), basipetal (+ 0.5), diffuse or divergent (+ 1.0)
(8)	Expansion	No expanded (flat/ ascidiate) growth (− 0.5), expanded (flat/ ascidiate) growth (+ 0.5)
(9)	Phyllotaxis^a^	No branching (− 1.0), spiral/alternate incl. monostichous and distichous (− 0.5), opposite/verticillate and multijugate (0.5), irregular (+ 1.0)
(10)	Branching (origin of branches from element)^b^	No branching (− 0.5), exogenous branching (0.0), endogenous branching (0.5)
(11)	Vascular tissue distribution	Xylem and phloem in alternating sectors or scattered (− 0.5), xylem and phloem in same axial sectors/collateral and conjoint (+ 0.5)

^a^Modified from Jeune et al. (2006)

^b^As described by Jeune and Sattler (1992)

**Table 4:** Data matrix of developmental processes and their values in a selection of typical roots, shoots and leaves, in the root of *P. moranensis*, and in vegetative organs of *U. dichotoma*

Organ	Modalities and values										
	1	2	3	4	5	6	7	8	9	10	11
LC1	1.0	1.0	− 1.0	− 1.0	0.5	0.5	− 0.5	0.5	− 0.5	0.0	0.5
LC2	1.0	1.0	− 1.0	− 1.0	0.5	0.5	− 0.5	0.5	0.5	0.0	0.5
LC3	1.0	1.0	− 1.0	− 1.0	0.5	0.5	0.5	0.5	− 0.5	0.0	0.5
LC4	1.0	1.0	− 1.0	− 1.0	0.5	0.5	0.5	0.5	0.5	0.0	0.5
LC5	1.0	1.0	− 1.0	0.0	0.5	0.5	− 0.5	0.5	− 0.5	0.0	0.5
LC6	1.0	1.0	− 1.0	0.0	0.5	0.5	− 0.5	0.5	0.5	0.0	0.5
LC7	1.0	1.0	− 1.0	0.0	0.5	0.5	0.5	0.5	− 0.5	0.0	0.5
LC8	1.0	1.0	− 1.0	0.0	0.5	0.5	0.5	0.5	0.5	0.0	0.5
LE1	1.0	1.0	− 1.0	− 1.0	0.5	0.5	− 0.5	0.5	− 1.0	− 0.5	0.5
LE2	1.0	1.0	− 1.0	− 1.0	0.5	0.5	0.5	0.5	− 1.0	− 0.5	0.5
LE3	1.0	1.0	− 1.0	0.0	0.5	0.5	− 0.5	0.5	− 1.0	− 0.5	0.5
LE4	1.0	1.0	− 1.0	0.0	0.5	0.5	0.5	0.5	− 1.0	− 0.5	0.5
SR1	0.5	− 1.0	− 1.0	− 1.0	0.5	− 0.5	− 0.5	− 0.5	− 0.5	0.0	0.5
SR2	0.5	− 1.0	− 1.0	− 1.0	0.5	− 0.5	− 0.5	− 0.5	0.5	0.0	0.5
SR3	0.5	− 1.0	− 1.0	0.0	0.5	− 0.5	− 0.5	− 0.5	− 0.5	0.0	0.5
SR4	0.5	− 1.0	− 1.0	0.0	0.5	− 0.5	− 0.5	− 0.5	0.5	0.0	0.5
SC1	0.5	− 1.0	− 1.0	− 1.0	− 0.5	0.5	− 0.5	− 0.5	− 0.5	0.0	0.5
SC2	0.5	− 1.0	− 1.0	− 1.0	− 0.5	0.5	− 0.5	− 0.5	0.5	0.0	0.5
SC3	0.5	− 1.0	− 1.0	0.0	− 0.5	0.5	− 0.5	− 0.5	− 0.5	0.0	0.5
SC4	0.5	− 1.0	− 1.0	0.0	− 0.5	0.5	− 0.5	− 0.5	0.5	0.0	0.5
SD1	0.5	− 1.0	− 1.0	− 1.0	0.5	0.5	− 0.5	− 0.5	− 0.5	0.0	0.5
SD2	0.5	− 1.0	− 1.0	− 1.0	0.5	0.5	− 0.5	− 0.5	0.5	0.0	0.5
SD3	0.5	− 1.0	− 1.0	0.0	0.5	0.5	− 0.5	− 0.5	− 0.5	0.0	0.5
SD4	0.5	− 1.0	− 1.0	0.0	0.5	0.5	− 0.5	− 0.5	0.5	0.0	0.5
TR	− 1.0	− 1.0	1.0	1.0	0.5	− 0.5	− 1.0	− 0.5	1.0	0.5	− 0.5
PR	− 1.0	− 1.0	− 1.0	1.0	0.5	− 0.5	− 0.5	− 0.5	1.0	− 0.5	− 0.5
I	− 0.5	1.0	− 1.0	− 1.0	0.5	− 0.5	− 0.5	− 0.5	0.5	0.0	0.5
Rp	− 1.0	− 1.0	− 1.0	0.0	0.5	0.5	− 0.5	− 0.5	− 0.5	0.0	− 0.5
Ra	0.5	− 1.0	− 1.0	0.0	0.5	0.5	− 0.5	− 0.5	− 0.5	0.0	− 0.5
s	0.5	1.0	− 1.0	1.0	0.5	0.5	− 0.5	0.5	− 0.5	0.0	− 0.5
a	0.5	1.0	− 1.0	1.0	0.5	0.5	− 0.5	− 0.5	− 1.0	− 0.5	− 0.5
Lp	1.0	1.0	− 1.0	− 1.0	0.5	0.5	0.5	0.5	− 1.0	− 0.5	− 0.5
La	0.5	1.0	− 1.0	− 1.0	0.5	0.5	0.5	0.5	− 1.0	− 0.5	− 0.5
bp	1.0	1.0	− 1.0	1.0	0.5	0.5	− 0.5	0.5	− 1.0	− 0.5	− 0.5
ba	0.5	1.0	− 1.0	1.0	0.5	0.5	− 0.5	0.5	− 1.0	− 0.5	− 0.5

Number of modalities (cf. Table 3 for explanations of values): 1 = positioning, 2 = growth period, 3 = orientation, 4 = geotropism, 5 = developmental symmetry, 6 = final symmetry, 7 = growth distribution, 8 = expansion, 9 = phyllotaxis, 10 = branching, 11 = vascular tissue distribution. Values are based on data from Jeune and Sattler (1992) and on results from the present paper

*LC* typical compound leaf, *LE* typical entire leaf, *SR* typical radial shoot/stem, *SC* typical shoot with changing symmetry, *SD* typical dorsiventral shoot, *TR* typical root, *PR* root of *P. moranensis*, *I* inflorescence (whole shoot), *Rp* primary runner stolon, *Ra* axillary runner stolon, *s* simple stolon, *a* anchor stolon, *Lp* first leaf on node, *La* axillary leaf, *bp* first bladder on node (flanking Lp), *ba* axillary bladder

The set of modalities (outlined in Table 3) was mainly compiled from those used in Jeune and Sattler (1992) and Jeune et al. (2006), and a range from − 1.0 to 1.0 or − 0.5 to 0.5 was applied for their values, following Jeune and Sattler (1992). However, to avoid complete congruence of structures in the morphospace of *U. dichotoma*, the set of modalities was supplemented with processes for the growth of expanded (flat or ascidiate) structures and the distribution of vascular tissue (Table 3: modalities 8 and 11). Modality 8 represents the growth of flat or ascidiate structure from an organ, generally expanding laterally/marginally or on a transversal zone (‘Querzone’). It was added since expanded forms are not necessarily restricted to dorsiventrality (cf. Tsukaya 2014), and some cylindrical forms can have a dorsiventral organization (cf. Rutishauser 2016). Modality 11 considers the distributional patterns of xylem and phloem that typically differ between roots and shoots, with roots having vascular tissues often in alternating sectors, and shoots showing collateral and conjoint vascular tissues in the same sectors (Groff and Kaplan 1988), whilst Sattler and Jeune (1992) used the static parameter of the presence/absence of vascular tissue.

We ran a Pearson correlation-type PCA with the XLSTAT statistical and data analysis solution, Addinsoft (2019), Boston, USA (https://www.xlstat.com), with data as listed in Table 4.

## Results

### Early development of the stolon and stolon nodes

**Fig. 1:**
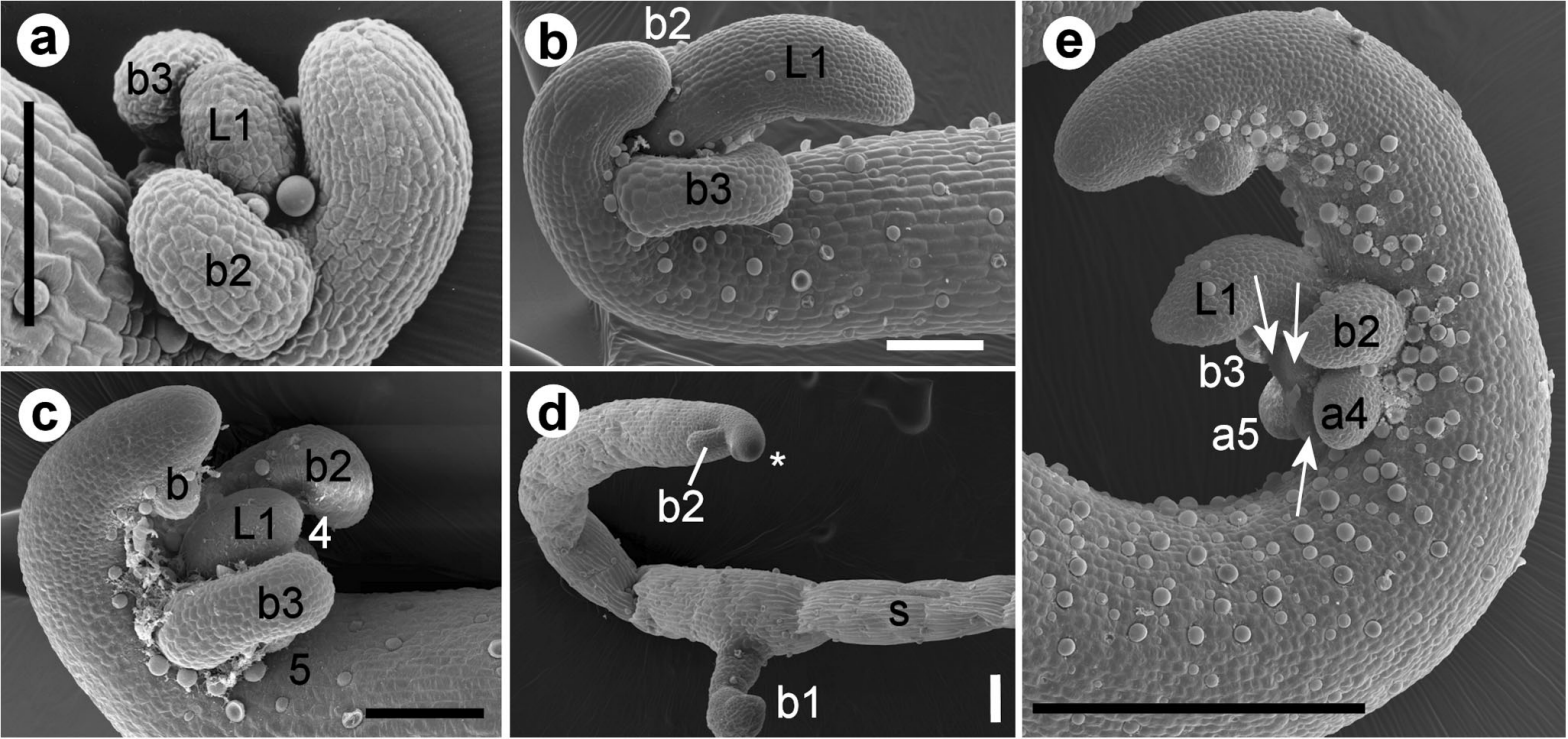
Early development of organs on stolons of *U. dichotoma*. **a** Stolon tip on a plant from Strathgordon, Tasmania, with the first foliage leaf (L1) and two flanking bladders (b2–b3) arising nearly simultaneously and initiating a stolon node. **b** Stolon tip on a cultivated plant from New Zealand showing the first leaf (L1) growing faster than the bladders (b2–b3). **c** Stolon tip on a plant from Bog Inn, Pureora Forest, New Zealand, with the bladders (b2–b3) growing faster than the first leaf (L1); primordia 4 and 5 are barely visible; a new bladder primordium develops on the next node at the stolon tip. **d** Simple stolon (s) of *U. dichotoma* from Anglesea, Victoria, Australia, with a juvenile bladder (b1) and a bladder primordium (b2) close to the stolon tip (star). **e** Stolon of the cultivated ‘robust clone’ from Newcastle, NSW, Australia, showing a node with a leaf (L1) and two flanking bladders (b2–b3); a rosette with primordia of two anchor stolons (a4–a5) and three additional primordia (arrows) develops in central and proximal position at the leaf base (same tip as in Fig. 2d). Numbers of organs/primordia represent the rank in the developmental sequence. Scale bar is 0.1 mm in **a**–**c** and 0.3 mm in **d**–**e**

**Fig. 2:**
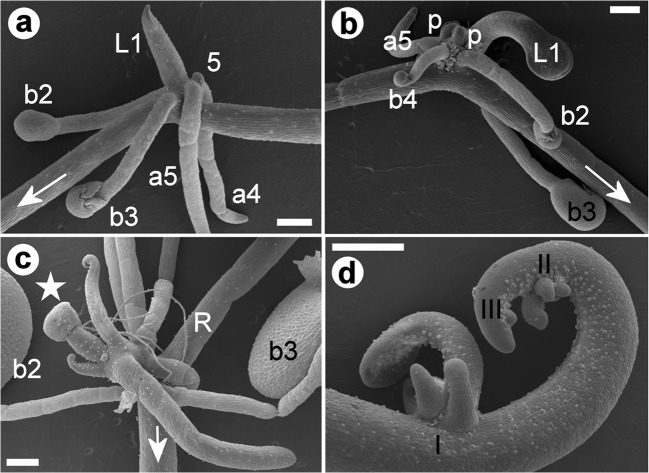
Further development of organs on stolon nodes, and elongation of stolons of *U. dichotoma*. **a** Stolon node of a plant from Bog Inn, Pureora Forest, New Zealand, showing the common pattern with a first foliage leaf (L1), two juvenile bladders (b2–b3), and a rosette with two anchor stolons (a4–a5) and a primordium (5) of unknown developmental fate. **b** Stolon node of a plant from the Kopouatai Peat Dome, New Zealand, showing the first leaf (L1) and three bladders (b2, b3 and b4), an anchor stolon (a5), and two primordia (p). **c** Runner stolon (R) of a plant from Lake Ohia, New Zealand, with two bladders (b2–b3) and a rosette of organs terminating with an inflorescence (star). The first foliage leaf is missing or was detached; other organs cannot be determined. **d** Cultivated ‘robust clone’ from Newcastle, NSW, Australia, showing the succession of nodes I–III along the elongating stolon. A close-up of nodes II and III is shown in Fig. 1e. Numbers of organs/primordia represent the rank in the developmental sequence. The arrows point to the direction of the stolon tip. Scale bar is 0.3 mm

**Fig. 3:**
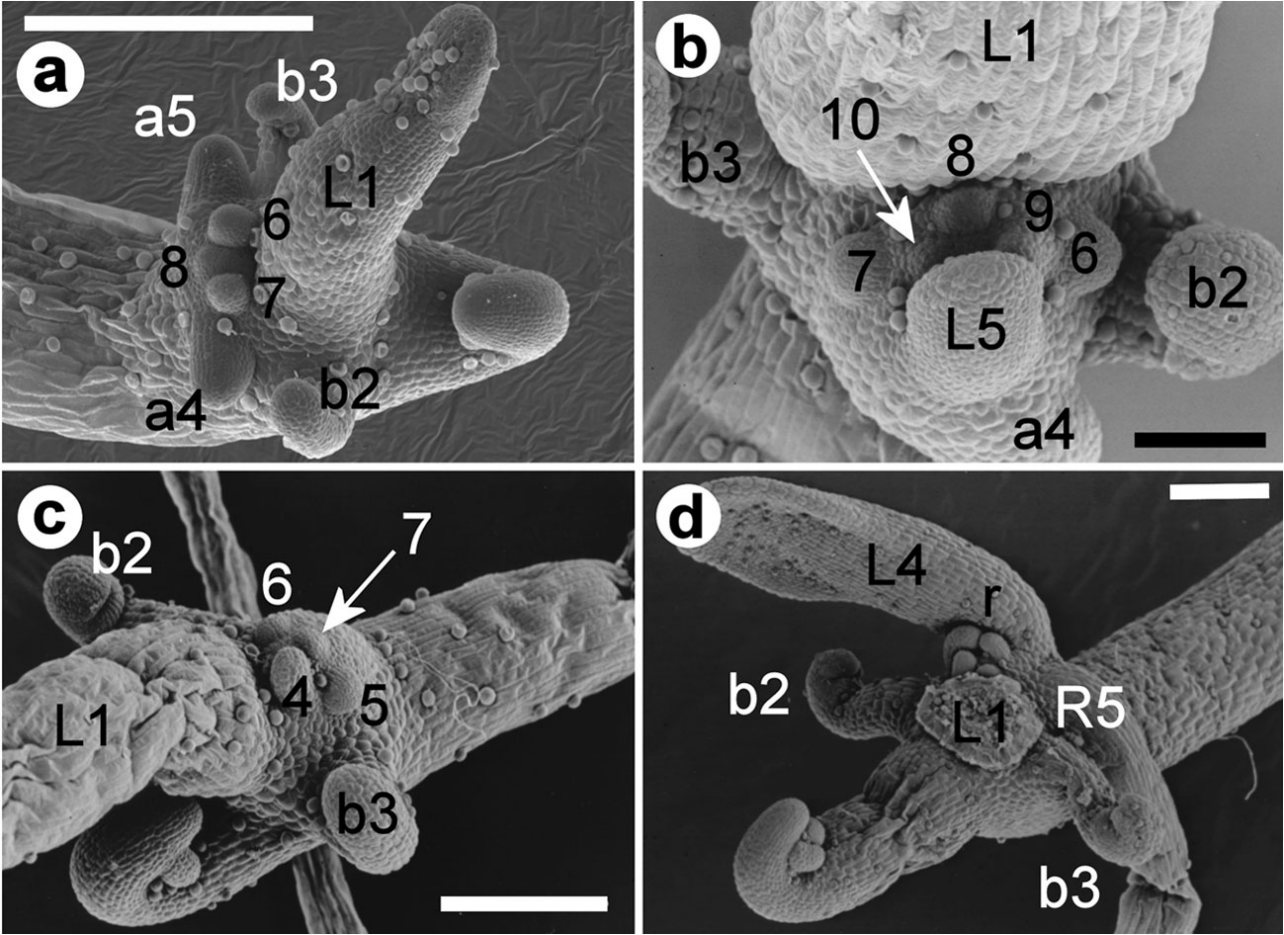
Organotaxis of rosettes on stolon nodes of *U. dichotoma* ‘Chestnut’ from Victoria, Australia (**a**), and of *U. dichotoma* from Strathgordon, Tasmania (**b**–**d**). Initiation of stolon nodes of **a**–**d** is with a first foliage leaf (L1) and two bladders (b2–b3). **a** Rosette of two anchor stolons and three primordia (6–8). **b** Rosette of an anchor stolon (a4), a leaf (L5) and five primordia (6–10). **c** Rosette of four primordia (4–7). **d** Rosette of two leaves (L1 = removed, L4), a runner stolon (R5), and four primordia (r) in decentralized position at the leaf base. Numbers of organs/primordia represent the rank in the developmental sequence. Scale bar is 0.3 mm for **a**, 0.1 mm for **b** and 0.2 mm for **c**–**d**

The early development of stolons was examined on all 24 sources/populations of *Utricularia dichotoma*. Runner stolons arose either from the primary shoot or from a runner stolon node. The first developmental stage of a stolon tip was characterized by a suppressed longitudinal growth of the upper meristematic cells. This led to a crozier like stolon tip, bent upwards and backwards (Fig. 1a–c). On the inside of the curled zone and close to the stolon tip, three primordia developed, initiating a so-called node. They arose exogenously and almost simultaneously on the upper side of the stolon (Fig. 1a–c). The central primordium grew slightly later and apparently became the first foliage leaf on the node as it soon dorsiventrally flattened in somewhat oblique-transverse orientation (Fig. 1a). The other two primordia were on either side of the leaf primordium but a bit more proximal. They longitudinally flattened and had a slightly curled appearance in a later developmental stage (Fig. 1a–c). During further growth, the distal part of each of them formed a peltate-ascidiate structure (a bladder), while the basal part elongated, bent downwards, uncurled and developed into the stalk (petiole) of the bladder (Fig. 2b). The growth axis of these two bladders was almost transverse to the axis of the runner stolon (Fig. 3a–d). During longitudinal growth of the runner stolon, the first leaf often grew more rapidly than the bladders (Fig. 1b, e), but in some cases, the development was retarded (Fig. 1c).

The development of organs was studied on 324 nodes from 22 sources/populations of *U. dichotoma*. The full data set with all developmental patterns found is available upon request. Only the condition of the plant material from Stewart Island, New Zealand, and from the Grampians, Victoria, Australia, was not suitable to identify developmental patterns on stolon nodes. Furthermore, plants from B.J. Płachno and K. Pasek 333 (cf. Table 1) were not included in the data set, since they were represented by their cultivated clones M.S. Reut 101 and M.S. Reut 102 (cf. Table 1). Around 30% of the nodes deviated from the common initial pattern of one leaf and two bladders, originating from primordia rank 1–3. Occasionally (4%) the node lacked the presence of a foliage leaf, but more often (21%), one of the bladders was absent or reduced. In most cases, however, the insertion or base of the missing organ was still apparent, indicating that the organ was already dropped or detached during preparation of the material. Only the material from Queenstown, Tasmania, and from Katoomba, NSW, Australia, showed no typical initial pattern, but the number of nodes examined was very limited (2 and 1, respectively).

### Organotaxis on stolon nodes

**Fig. 4:**
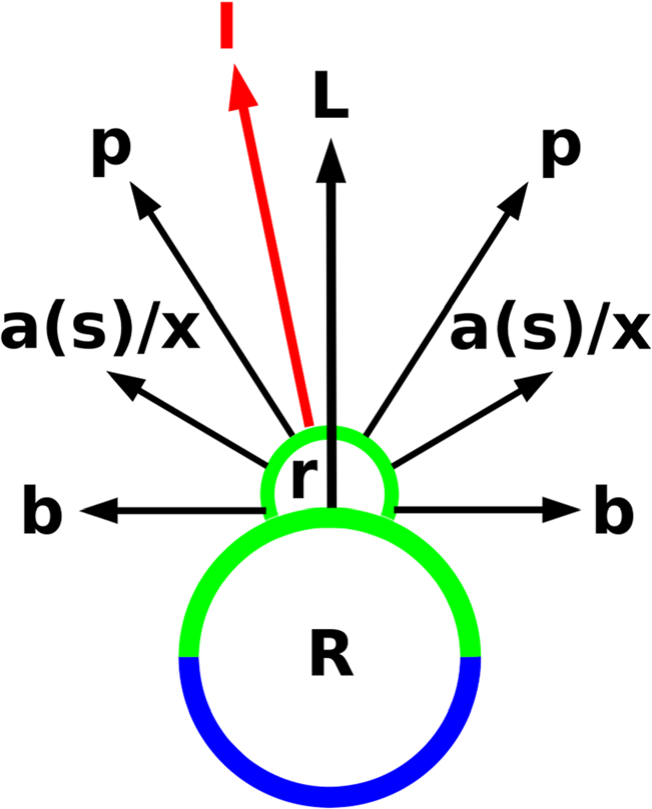
Generalized pattern of stolon branching in *U. dichotoma*. On the upper sector of the runner stolon (R; green = upper/adaxial sector, blue = lower/abaxial sector), after the development of a first foliage leaf (L) and two bladders (b), organs grow from the base of the leaf in a rosette (r). a(s)/x = develops into an anchor stolon (a) which may turn into a simple stolon (s) or develops (less often) into another organ type (x) such as a bladder, a leaf, or a runner stolon at the same insertion. Primordia (p; organ rank 6 or higher) develop into a, s, b, L, or R. I = inflorescence (red)

On the proximal side of the first leaf, on a swollen base, up to seven further primordia (rank 4–10) developed exogenously (Figs. 2a, 3a-d and 4). They formed a rosette of various possible combinations of anchor or simple stolons, runner stolons, bladders and foliage leaves.

Although generally positioned centrally on the proximal side of the first leaf, slightly displaced rosettes were found, even on stolons of the same plants (Fig. 3d). Occasionally, nodes bore a short stem of a rosette with several (3–6) leaves and other organs. On the top of the rosette, eventually an inflorescence (Fig. 2c) was produced, which developed numerous anchor stolons around the base of the peduncle and above all other organs of the rosette.

A consistent developmental order and phyllotaxis of organs of the rosette could not be clearly identified; it seemed to be irregular but often centripetal and presumably distichous (Fig. 3a–d). However, in several rosettes, the first two of the primordia (i.e. rank 4 and 5) grew seemingly simultaneously in more proximal position and faster than the primordia closer to the first leaf (Fig. 3a, b, d).

During differentiation of the organs on the stolon node, the runner stolon uncurled and underwent a monopodial, longitudinal growth. At that time, an equal organ development had already been initiated at the stolon tip to form a second node (Fig. 1e). Curling, node development and longitudinal growth were repeated several times (Fig. 2d). This way, the stolon elongated, and the plant (colony) spread.

### Organ combinations on stolon nodes

**Fig. 5:**
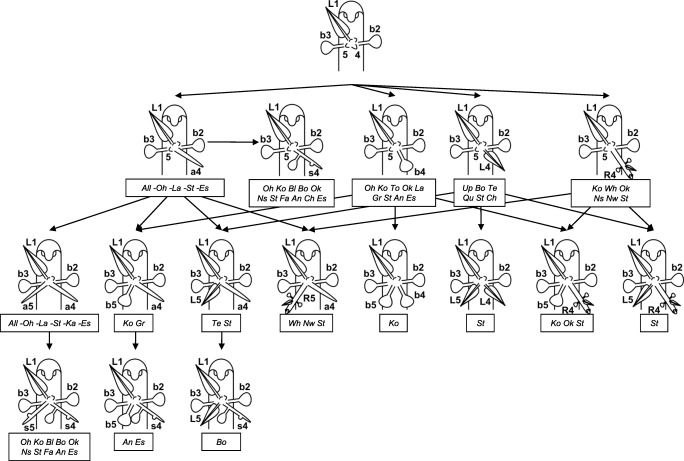
Most abundant patterns of organ combinations in position 4 and 5 of rosettes on a runner stolon node in *U. dichotoma*. After development of a first foliage leaf (L1) and two bladders (b2-b3), a rosette with next primordia (4–5) was built at the base of the leaf. The first organ of the rosette was either an anchor stolon (a4), a bladder (b4), a leaf (L4), or a runner stolon (R4). The sequence continues with the development of an anchor stolon (a5), a bladder (b5), a leaf (L5), or a runner stolon (R5). During further elongation, anchor stolons may build bladders; in this form, they are called ‘simple stolons’ (s). Numbers of organs/primordia represent the rank in the developmental sequence. Organs of ranks 4 and 5 may be inverted. Populations/sources (cf. Table 1 for abbreviations), in which the organ combinations of rank 4–5 were found, are shown in boxes in italic, whereas, e.g. ‘all -Oh, -La, etc.’ means that this combination was found in all populations except for the material from Lake Ohio (Oh), Lake Pearson (La), etc. Note that there may be more sources shown for a specific organ in rank 4 than sources for rank 4 and 5 in sum, since they may have contained nodes with unidentifiable organs after position 4

From the 324 nodes investigated across 22 sources/populations, 111 organ combinations were recorded. A consolidation of patterns revealed 11 combinations of organs originating from primordia 4 and 5 as illustrated in Fig. 5, although organs from primordia 4 and 5 may also appear in inversed position.

In the majority of populations, primordium 4 and 5 generally developed into anchor stolons (Figs. 1e, 2a and 3a). Patterns with runner stolons and/or leaves arising from primordium 4 and 5 were seen in several populations but predominantly in samples from Strathgordon, Tasmania (Fig. 3b, d). The latter was the only population showing a combination of a runner stolon with a leaf (Fig. 3d), and a combination of two leaves (Fig. 5). Most stolon rosettes from the Kopouatai Peat Dome, New Zealand, showed one or two bladders originating from these primordia (e.g. a bladder and an anchor stolon as in Fig. 2b). One bladder on position 4 or 5 occurred also in samples from several other sources from Australia and New Zealand, although less abundantly, but two bladders on position 4 and 5 were a unique pattern across the 22 populations/sources investigated (Fig. 5).

Combinations of organs deriving from primordia 6–10 of the rosette were greatly variable, and a clear pattern could not be identified. Ten out of 22 populations had branched stolons, i.e. rosettes producing secondary runner stolons. Additional anchor stolons and simple stolons were rare in rank 6–10 and observed mainly on the base of peduncles. In some populations, the development of additional (2–5) foliage leaves and other organs led to a slight prolongation of the rosette to form a short stem (e.g. in New Zealand plants from the cultivation experiment, and in plants from Falls Creek, Victoria, Australia).

Plants from the Kopouatai Peat Dome, New Zealand, showed unique rosettes with up to five bladders in a whorl, and occasionally a second whorl with bladders and runner stolons on top of the former whorl.

Simple stolons were recorded from 11 of 22 populations, in which they generally developed on mature nodes and grew up to several centimetres. However, they were also observed in samples from Te Kauwhata, New Zealand, and from Queenstown, Tasmania, but they could not be assigned to stolon nodes due to the sparse material. Simple stolons from these two sites were, therefore, not included in Fig. 5. Simple stolons occurred predominantly on nodes from plants of Falls Creek, Victoria, Australia. The shorter anchor stolons rarely co-occurred with simple stolons on the same node. However, simple stolons were also noticed on peduncles of plants from the Grampians, Victoria, Australia, alongside with numerous anchor stolons. Simple stolons carried up to 3, rarely 4, bladders in a row (Fig. 1d). Only the most mature bladders, i.e. those proximal to the stolon node, contained organisms and soil matter.

In the cultivation experiment, plants in the wet soil showed more short stems of rosettes with leaves than plants in the water-logged soil, hence more leaves overall. In the same experimental setup, simple stolons were much more abundant on plants in water saturated substrate than on plants in wet soil. The length of internodes did not differ significantly between plants of these two clones.

### Anatomy of vegetative organs

**Fig. 6:**
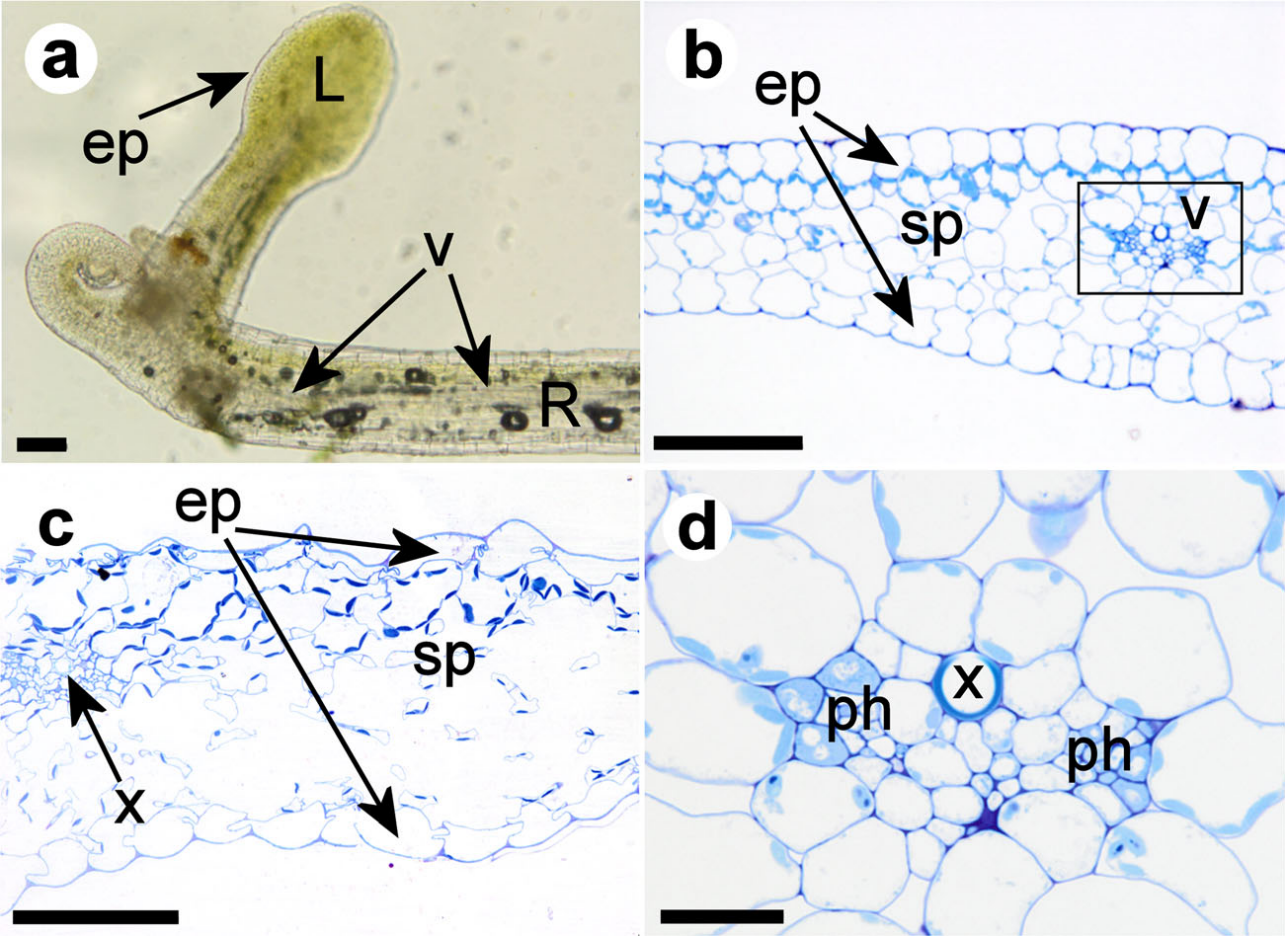
Anatomy of the stolon tip and the leaf of *U. dichotoma*. **a** Longitudinal view through a stolon of *U. dichotoma* from Katoomba, NSW, Australia, showing photosynthetically active tissue below the epidermis (ep) of the foliage leaf (L) and on the upper side of the runner stolon (R). One vascular strand (v) is visible within the runner stolon. **b**–**d** Cross sections through foliage leaves. **b** Plant from Newcastle, NSW, Australia, with an epidermis (ep) on the upper and lower side of the leaf, spongy parenchyma (sp), and a vascular strand (v). Details of the vascular tissue (in the rectangle) are outlined in **d**. **c** Plant from New Zealand with spongy parenchyma (sp) and one vascular strand consisting of one tracheary xylem element (x). The epidermal cells (ep) of the upper side of the leaf are more irregular in shape than the ones of the lower side. The irregular shape of the cells of the upper leaf epidermis is probably caused by tissue shrinkage prior or during the fixation process of the specimen. **d** Details of the vascular strand of picture b showing one tracheary xylem element (x) and at least two distinctive phloem tissues (ph). In leaves of **b** and **c** chloroplasts are more abundant in parenchyma cells of the upper leaf side. Scale bar is 0.1 mm in **a**–**c** and 0.02 mm in **d**

**Fig. 7:**
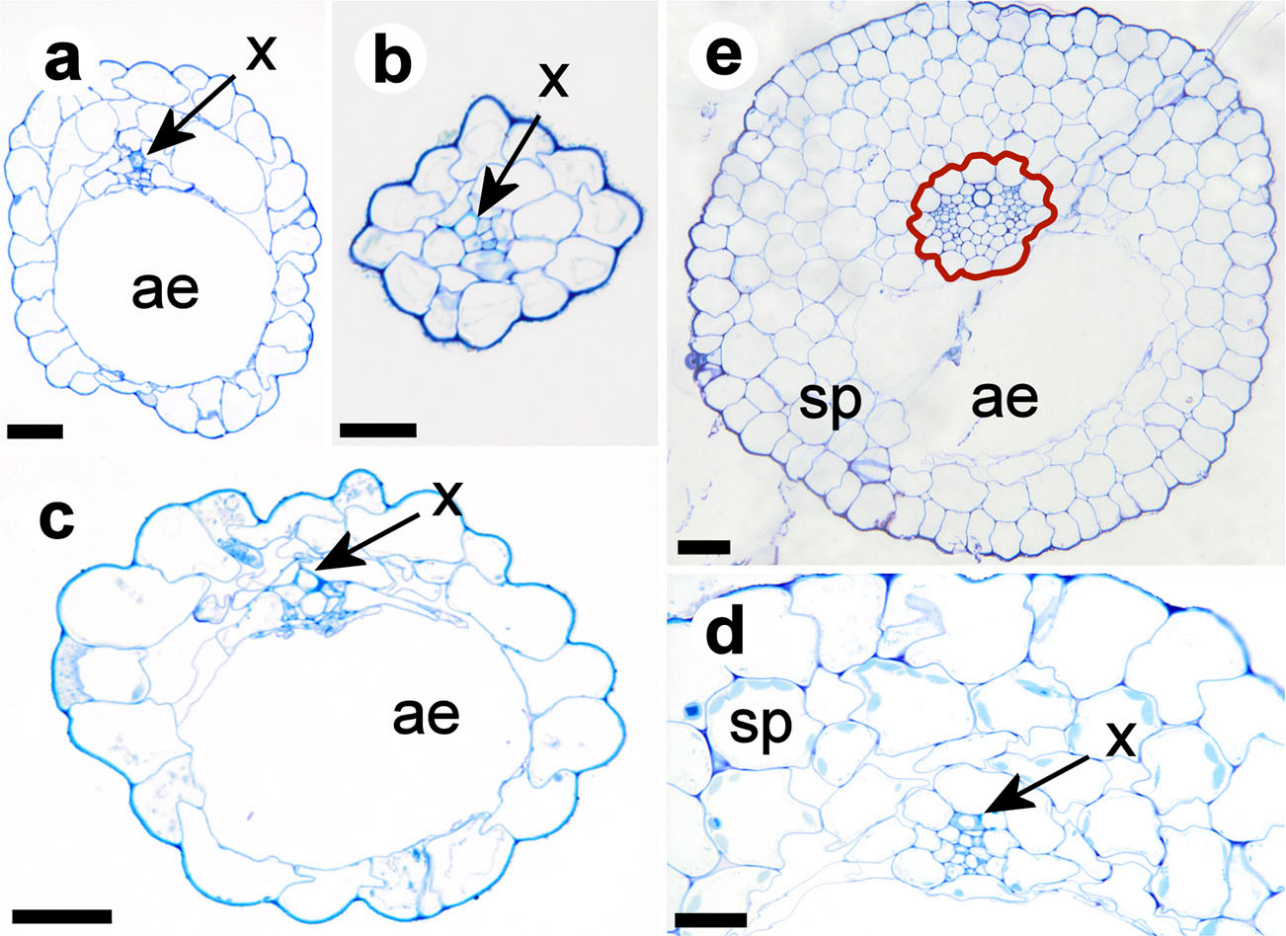
Cross sections through stolons and bladder stalks of *U. dichotoma* with spongy parenchyma (sp), aerenchyma (ae) and tracheary xylem elements (x). **a** Anchor stolon of a plant from New Zealand. **b** Apical region of an anchor stolon of the alpine form from Falls Creek, Victoria, Australia. **c** Trap stalk of a plant from New Zealand. **d** Close-up of a trap stalk of a plant from Newcastle, NSW, Australia. **e** Runner stolon of a plant from Newcastle, NSW, Australia. The vascular bundle is completely surrounded by parenchyma cells, visualized by the red borderline. Details are shown in Fig. 8d. Scale bar is 0.02 mm in **a**–**d** and 0.05 mm in **e**

**Fig. 8:**
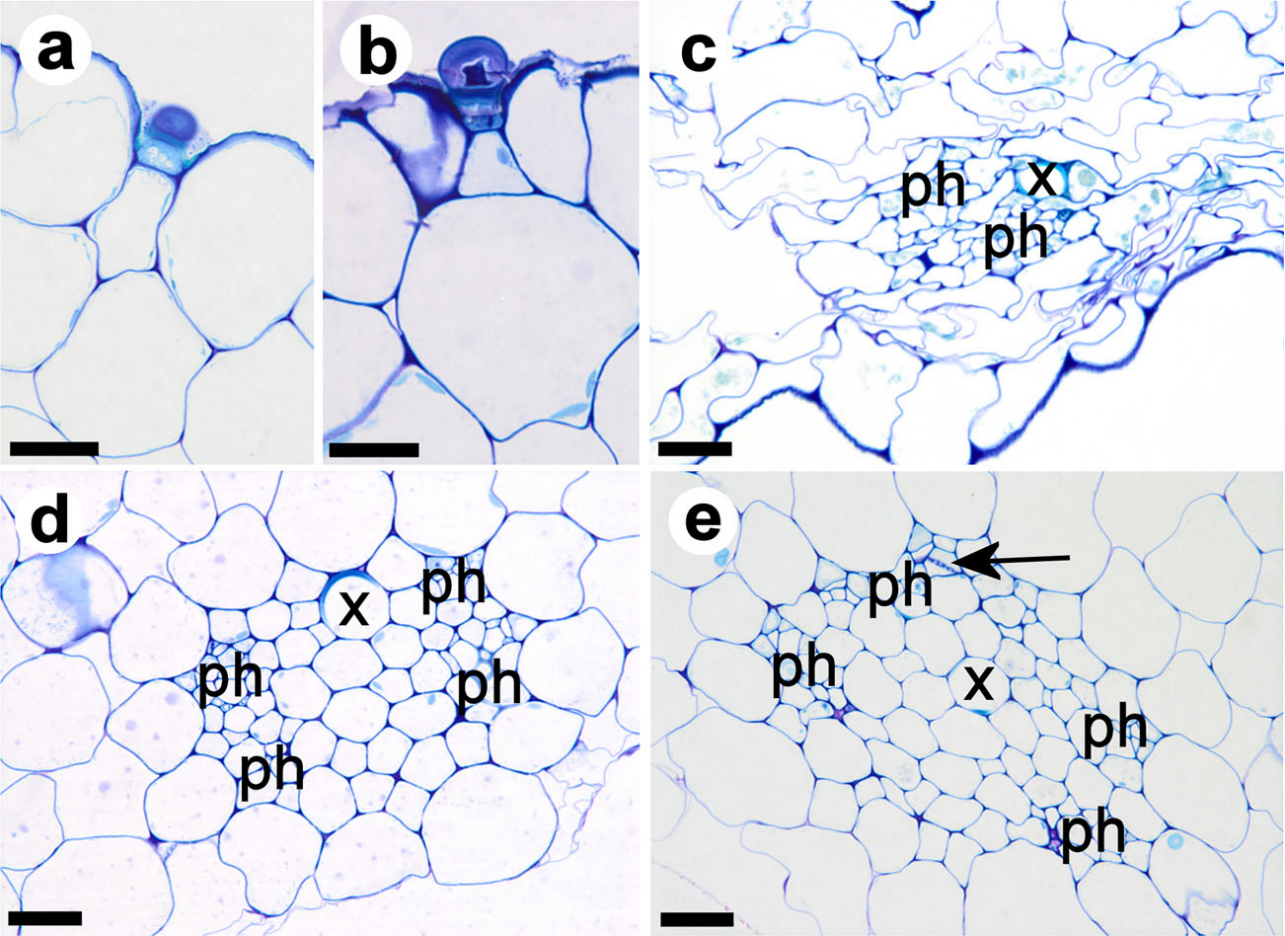
Glandular and vascular tissue of runner stolons of *U. dichotoma* from Newcastle, NSW, Australia (**a**, **b**, **d**, **e**) and New Zealand (**c**). **a**, **b** Glandular (button-like) trichomes, more or less sunken in the epidermis. **c**–**e** Vascular bundles with xylem element (x) and phloem groups (ph). **c** Tissues look irregular or squeezed, and phloem elements are difficult to identify. **d** Detail from Fig. 7e showing one xylem element and four phloem groups. **e** Vascular bundle with one xylem element (x) and four phloem groups (ph). The arrow points to a sieve plate. Scale bar is 0.02 mm

The anatomy of foliage leaves, runner stolons, anchor stolons and petioles of bladders was investigated on five sources of *Utricularia dichotoma* from Victoria, New South Wales and New Zealand. Histological structures were laid out dorsiventrally. They did not differ significantly between the organ types and between the sources (Figs. 6, 7 and 8).

Foliage leaves revealed an epidermis with a thin cuticle, a vascular bundle with one xylem tracheary element and up to two phloem groups, spongy parenchyma but no palisade parenchyma (Fig. 6a–d). Stolons and petioles showed a dominant air chamber on the lower sector, collapsed parenchyma, and a vascular tissue with one xylem tracheary element and indefinite phloem (in anchor stolons and petioles, Fig. 7a–d), or 2–4 phloem groups flanking the xylem on the lower side (in runner stolons, Fig. 8c–e). Chloroplasts were restricted to parenchyma cells on the adaxial side of leaves and upper side of runner stolons. Unicellular hairs were absent. Button-like trichomes were abundant on all types of vegetative organs (cf. Fig. 8a, b), but on runner stolons, they were more numerous and larger on the upper part of the distal region (Fig. 1b, c).

### Dynamic morphology of vegetative organs

**Fig. 9:**
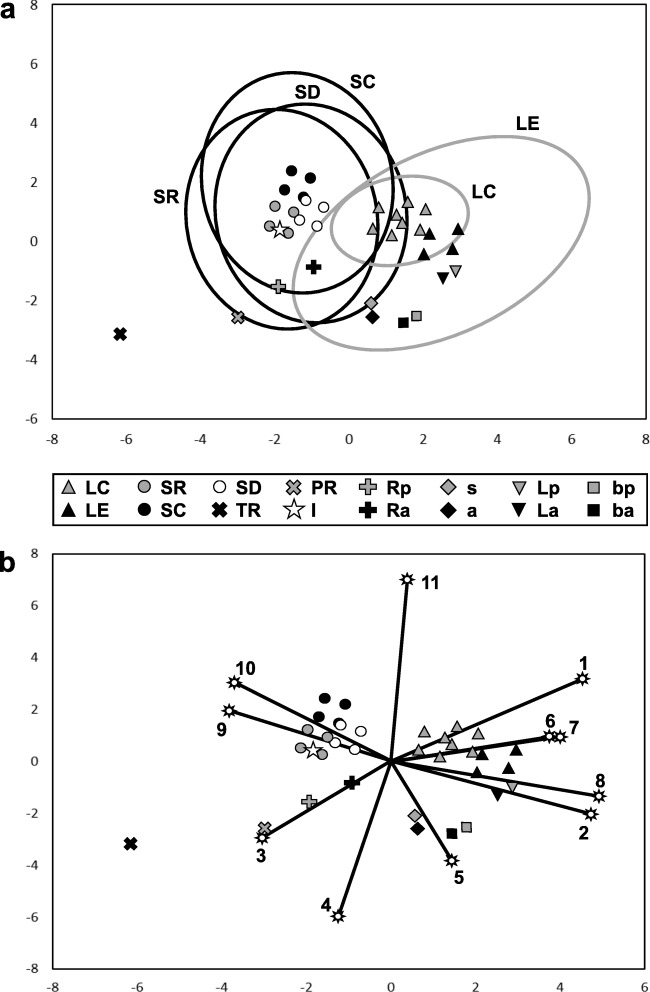
Principal component analysis of modalities (developmental processes) of vegetative organs of *U. dichotoma*, the root of *P. moranensis*, and a selection of typical roots, shoots and leaves. Principal component 1 (*x*-axis) is 37.34%; principal component 2 (*y*-axis) is 19.60%. **a** Morphospace of organs included in the PCA showing their distribution and distances (correlation) to each other (cf. Table 4 for abbreviations). Typical shoots and leaves are grouped (SR = SR1–4, SC = SC1–4, SD = SD1–4, LC = LC1–8, LE = LE1–4), and confidence ellipses (CI = 95%) of the groups are shown. **b** Distance biplot showing the distribution of organs in relation to modalities used in the PCA (see Table 3 for numbers of modalities and their meaning). Adjacent modalities show a positive correlation; opposite modalities indicate a negative correlation. Organs close to a modality axis are strongly influenced by the respective modality. A longer modality axis indicates that the modality has contributed more to the PCA.

The data matrix of Table 4 was complemented with the information from Jeune and Sattler (1992) on process combinations of the *Pinguicula moranensis* root and typical roots, shoots and leaves, and with findings from our morphological and anatomical investigations on *Utricularia dichotoma*. Results of the PCA are graphically illustrated in Fig. 9a, b. Based upon Fig. 9a, leaves, bladders, anchor stolon, simple stolon and the axillary runner stolon of *U. dichotoma* are correlated with typical entire leaves, i.e. distributed within their confidence ellipse, with leaves having the strongest and runners having the weakest association with typical entire leaves. Stolons of *U. dichotoma* combine processes for typical leaves and typical shoots, whilst runner stolons show the strongest correlation with typical shoots. The inflorescence axis is very strongly correlated with typical shoots. The root of *P. moranensis* and the mother runner stolon of *U. dichotoma* are the closest organs to the typical root, but the latter is far outside the confidence ellipses of typical shoots and leaves. Based upon Fig. 9b, typical shoots are strongly associated with their developmental processes for (exogeneous) branching and phyllotaxis, and typical leaves with their (axillant) positioning, final (dorsiventral) symmetry, growth distribution, growth period and development of flat structures. A mixture of modalities mainly drives the development of the typical root to some extent, namely positioning (not in connection with shoot axils, and not a main axis), final (radial) symmetry and a mixed (basipetal and acropetal) growth distribution. In *U. dichotoma*, leaves are mostly influenced by their determinate growth period and growing a flat lamina. Anchor stolon, simple stolon and bladders of *U. dichotoma* seem to be mainly driven by their (non-changing) developmental symmetry, although this modality is the weakest in the data set. Runner stolons of *U. dichotoma* and the root of *P. moranensis* have a strong association with the modality ‘orientation’ (no cap produced), but they are also influenced by (non-axillant) positioning. The developmental processes for ‘geotropism’ and ‘vascular tissue distribution’ turned out to have less influence on the determination of organs, but they helped to separate some organs in the distribution chart.

## Discussion

### Vegetative morphology

The current study encompasses more sources from a broader geographic range than previously used for morphological investigations on *Utricularia dichotoma* or other species of subgenus *Polypompholyx* (cf. Reut and Fineran 2000). The initial development of a leaf and two bladders on the runner stolon was confirmed to be the most common pattern of the early stolon node development. It seems to be typical for the species. Only 2 out of 22 sources did not show this combination, but the number of stolon nodes studied from these two populations was very small. The initial pattern of a first foliage leaf and two flanking bladders seems to differ from other *Utricularia* species outside of subgenus *Polypompholyx*, although the initiation of further organs on the stolon node is on the same (proximal) side of the leaf in various members of subg. *Utricularia* such as *U. longifolia* and *U. sandersonii* (Brugger and Rutishauser 1989; Rutishauser 2016).

In *U. dichotoma*, the development of organs from the stolon node and from rosettes is always restricted to the upper sector (Fig. 5). In several other terrestrial non-*Polypompholyx* species, branching is mainly on the upper sector, but may appear also laterally (Brugger and Rutishauser 1989). Developmental patterns of organs originating from a bud at the base of the first foliage leaf varied greatly within and between populations of *U. dichotoma*. Results show that merely every possible combination of foliage leaf, anchor stolon, runner stolon and bladder (trap) develops from primordium 4 and 5. Provided that simple stolons are formed from anchor stolons, the maximum number of possible organ combinations from primordium 4 and 5 is 14. From this, only three combinations were missing in the material investigated: the combination of two runner stolons, a bladder with a leaf and a simple stolon with a runner stolon. Since the pattern of an anchor stolon with a runner stolon does occur (as also described by Lloyd 1942), it seems likely that the combination of a simple stolon with a runner stolon would exist in *U. dichotoma* as well.

The combination of two bladders at position 4 and 5 seems to be restricted to plants from Kopouatai Peat Dome, New Zealand. Another exclusive pattern in this population, which was already highlighted by Reut and Fineran (2000), is the production of up to five bladders in a whorl on the runner stolon. Similarly, whorls of three bladders were described by Merl (1915) on stolons of *U. volubilis* R.Br. of the same section *Pleiochasia*. Taylor (1989) and Lloyd (1942) observed the same whorls of three bladders in *U. volubilis* but referred to these stolons as rhizoids or anchor stolons, respectively. Exclusively on the plants of Kopouatai, the rosettes even occasionally grow second rosettes with bladders and runner stolons on top of the first whorl. Within subgenus *Polypompholyx*, verticillate branching has only been observed in the aquatic *U. tubulata*, which displays whorls of leaves and bladders on the lower part of its peduncle (Taylor 1989, pp. 138–139). Plants from Strathgordon, Tasmania, were found to be the only ones with two leaves, or a leaf and a runner stolon growing from primordium 4 and 5. Branching with secondary stolons and leaves is generally more abundant in this population than in plants from other sources.

In most populations, however, the initial growth of a foliage leaf and two bladders is followed by the development of a pair of anchor stolons from primordia 4 and 5, a pattern also illustrated by Lloyd 1942 (Figs. 19 and 20) and reported as abundant by Reut and Fineran 2000. Within populations, except for those of Kopouatai and Strathgordon, this pattern (along with the derived pair of simple stolons) was also the most common. The cultivation experiment and observations on several populations revealed that on mature nodes, anchor stolons may extend their longitudinal growth and produce bladders along their axis. This growth form is referred to as ‘simple stolons’, which seems to be expressed when the plants are more submerged. However, due to the absence of simple stolons in 9 out of 22 sources examined, the ability to grow simple stolons may as well be genetically determined. Functionally, simple stolons may take up nutrients by their mature bladders via decomposed organisms, or via external trichomes.

Patterns of organs of rank 4 and 5 occur usually in plants from both Australia and New Zealand. Although the combination of a bladder with an anchor stolon was found only in New Zealand plants (Kopouatai and the Groynes), and the combination of a bladder with a simple stolon was restricted to Australian material (Anglesea and Esperance), it can be expected that both patterns occur in both regions, considering that simple stolons are modified anchor stolons, and that Kopouatai material contains simple stolons. Similarly, the combination of a simple stolon with a leaf was found only in material of Bog Inn, Pureora Forest, New Zealand, but patterns with an anchor stolon and a leaf occurred in Australia and New Zealand, hence we can anticipate that a combination of a simple stolon with a leaf exists in Australian populations.

The results demonstrate that there is no exclusivity of organs of rank 4 (or inverted in rank 5) with respect to plants from cultivations vs. plants from their natural sites. However, for validating the observed patterns, further cultivation experiments on more ex-situ material from a variety of original sources would be useful, by considering different water levels and other factors. Alternatively, in situ material from several sites could be studied and compared to the same source material after cultivation.

In accordance with Lloyd’s observation (1942) on *U. dichotoma*, juvenile inflorescences are found, originating from the rosette at the base of the first foliage leaf on a stolon node. The development of inflorescences from the upper stolon sector coincides with the general habit of stoloniferous bladderworts (Rutishauser and Isler 2001). Anchor stolons, which are developed additionally on the peduncle base, serve to stabilize the inflorescence in the substrate.

### Anatomy

The delicate and small nature of *Utricularia dichotoma* and its vegetative organs correspond with a simple anatomy and a low number of vascular tissues (especially xylem tracheary elements). Numbers of phloem groups and parenchyma cell layers seem to increase with the larger size (diameter) of organs. Merl (1915) outlined the anatomy of tissues in the foliage leaves of plants of *U. dichotoma* from Tasmania and found one vascular bundle with 2–3 tracheary xylem elements. Our results show a more reduced structure, but Merl’s material might have been from more robust plants with broader leaves. Similar conclusions have been drawn by Brugger and Rutishauser (1989) regarding more robust leaves in epiphytic bladderworts. Largely concordant with Lloyd’s description (1942) of *U. dichotoma*, we could show that stolons and petioles have a lacunate histological structure and one simple vascular bundle within some parenchyma. Although the vascular tissues are very low in numbers, they seem to be scatteredly situated towards the interior of the organ (like in *Pinguicula moranensis* and similar to typical roots) rather than adjacent in conjoint bundles (cf. Brugger and Rutishauser 1989). Air chambers are dominant in subterranean organs of *U. dichotoma*. Jung et al. (2008) reported that the aerenchyma of the aquatic *U. tenuicaulis* (syn. *U. australis* pro parte sensu Taylor 1989) is wheel-shaped. In *U. dichotoma*, stolons show one prominent air chamber only in the lower sector. Aerenchyma formation in *U. dichotoma* may have two basic advantages: (a) the prominent air chambers enable gas transport and buoyancy, especially when the plant is submerged; and (b) the investment into biomass is lower during dry periods and/or when less nutrients are available (cf. Evans 2003; Takahashi et al. 2014).

Although *U. dichotoma* is classified as terrestrial bladderwort (Taylor 1989), the reduced anatomical structures of its vegetative organs show a strong congruence with hydrophytes (cf. Arber 1920; Sculthorpe 1967; Krähmer 2016), which points to an adaptation to water-logging and temporary submergence in the natural habitats (e.g. at the edge of ponds, lakes or streams, bogs, roadside ditches), obviously due to similar environmental pressures (gas metabolism and nutrient uptake, especially in submerged conditions). Often plants that can successfully establish in stressful environments contain a small genome (Knight et al. 2005). This trend is also apparent in hydrophytes, but it is particularly evident in wetland plants such as *U. dichotoma* coping with habitats of fluctuating dry and submerged situations (cf. Hidalgo et al. 2015). Nutrient deficiency in the substrate or water may have been an additional factor accompanying the genome shrinking in Lentibulariaceae down to sizes of, e.g. 246 Mbp in *U. dichotoma*, ~ 100 Mbp in *U. gibba*, and the ultrasmall ~ 61 Mbp in *Genlisea tuberosa* (cf. Fleischmann et al. 2014; Veleba et al. 2014; Wheeler and Carstens 2018).

As in many hydrophytes, the anatomy of typical roots of terrestrial angiosperms with, e.g. unicellular hairs, endodermis, a root cap and endogenous branching, is missing in *U. dichotoma*, but the plant has functionally alternative structures to take up nutrients. A reduced root system is also known from other carnivorous plants (Adlassnig et al. 2005). As reported from several aquatic and terrestrial bladderworts (cf. Heide-Jørgensen 1991), the cuticle of the epidermis of vegetative organs of *U. dichotoma* is thin but impermeable. Therefore, the uptake of nutrients from the water may be via external trichomes of subterranean organs (cf. Chormanski and Richards 2012), and from organic matter via internal quadrifids of the traps. Studies on the anatomy of vegetative organs in other species of subgenus *Polypompholyx* are underway (Reut and Płachno in prep.).

### Dynamic morphology

Goebel (1891) stated that the role of a plant part within the bauplan of a plant body cannot be solely determined by its anatomical characters. His view of *Utricularia* was primarily based upon observations of the development and position of organs in relation to each other, being it on the seedling or on shoots. Goebel (1891) defended the concept that all organs are leaves or transformed leaves, but he acknowledged that the prolonged apical growth of stolons does not fit into the classical understanding of an angiosperm leaf. However, we could compare the organogenesis of organs along the elongating runner stolon, with its monopodial growth, with the acropetal type of compound leaves development (cf. Tsukaya 2014). The dorsiventral organization with chloroplasts on the upper side of runner stolons in *U. dichotoma* would also account for a leaf-like character. With respect to terrestrial bladderworts, the conclusion of Troll and Dietz (1954) was contrary. To them, leaves were transformed shoots, i.e. phylloclades. Arber (1950) stated that leaves behave like whole-shoots and may at least be regarded as partial shoots.

In *U. dichotoma*, the initiation of a leaf and two bladders near the stolon tip, and in about one line and transverse to the stolon axis, is a special feature, in that these organs develop almost simultaneously. Thus, they could be interpreted as one compound palmate leaf, with the middle part growing to a seemly normal green leaf (which we called the first leaf), and the flanking organs developing into peltate-ascidiate leaves forming bladders. If the first leaf (or the whole compound palmate leaf) is regarded as subtending leaf, following classical morphology, we would expect that a shoot branches on the distal side of the subtending leaf. However, in *U. dichotoma*, the next organs, often anchor stolons, grow in the proximal axil of the first leaf, i.e. in invers-axillary position in relation to the stolon. The runner stolon can be interpreted as monostichous shoot similar as, e.g. in *Costus* (cf. Kirchoff and Rutishauser 1990), but with orthostichous, non-spiral phyllotaxis (i.e. organs arise always on the upper stolon sector with 0% divergence angle). In contrast, the runner stolon can be regarded as (compound) leaf with epiphyllous buds at the pinna nodes or along the midrib as, e.g. in *Chisocheton tenuis* (cf. Fisher and Rutishauser 1990) or *Mocquerysia* (cf. Dickinson 1978), respectively, although with only one leaflet (a compound palmate leaflet as described above) per node, and with invers-axillary branching of buds. Alternatively, as outlined by Rutishauser and Isler (2001), the runner stolon may be a ‘subtending shoot’ as intermediate step towards the ‘normal’ branching pattern (as evident in the inflorescence of *U. dichotoma*). Furthermore, *Utricularia* stolons have some anatomical similarities with roots, presumably as remnant of the relationship with *Pinguicula* (cf. Rutishauser and Isler 2001; Rutishauser 2016). Whichever hypothesis we assume in the example of the mother runner stolon, we end up with an architectural model that does not completely fit into the classical typology of organs. We need a different approach.

The results of the present study show that leaves, runner stolons, anchor stolons or bladders can develop in any combination on stolon nodes of *U. dichotoma*. Similar observations have been made by Brugger and Rutishauser (1989) on several terrestrial and epiphytic *Utricularia* species. Rutishauser and Isler (2001) concluded that the identity of various primordia in rosettes is not determined in an early stage. The interchangeability of these organ types on defined positions is an indicator for their homology or homeosis. The question would be: to which degree? In stolon rosettes of *U. dichotoma*, the primordia soon develop into a cylindric structure, which either continues to grow acropetally into an anchor stolon (and later eventually into a simple stolon), a runner stolon (with primordia of a leaf and two bladders close to its tip), a leaf (by flattening), or a bladder (by building an ascidiate structure on the apex).

Several genetic processes for the development of shoots, leaves and roots are responsible for the determination of the primordia in *Utricularia* (cf. Carretero-Paulet et al. 2015a). Figure 9a and b illustrates a quantitative approach of the degree of developmental process combinations for roots, shoots and leaves in all vegetative organs of the data set. The results indicate that leaves and bladders of *U. dichotoma* predominantly express processes for typical entire leaves. Stolons combine processes for typical leaves and typical shoots, with anchor stolons and simple stolons closer to typical leaves, and runner stolons closer to typical shoots. The inflorescence of *U. dichotoma* seems to represent a typical shoot. This coincides with the observation that the *Utricularia* inflorescence does not deviate from the normal bauplan with axillary branching (cf. Rutishauser 2016). Even if the correlation of vegetative organs of *U. dichotoma* with the typical root seems weak, it cannot be ruled out. The question of Brugger and Rutishauser (1989), whether the *P. moranensis* root could be interpreted as an intermediate form of a typical root and a typical shoot, can be answered in the affirmative by the results of our PCA. It matches as well with the analyses of Jeune and Sattler (1992: Figs. 1b, 3b) and Sattler and Jeune (1992: Figs. 2b and 3b). Despite the simplified anatomical structures in leaves, bladders and stolons of *U. dichotoma* with only few vascular elements, we assessed their distribution as closer to typical roots (modality 11, value − 0.5), since the elements occur more scatteredly in the centre of the organs. Even though *Utricularia* lacks a root with endogenous branching, unicellular hairs and root cap, it has been shown in the aquatic *U. gibba* and *U. vulgaris* that several genes participating in root development can still be found in the genome (Barta et al. 2015; Carretero-Paulet et al. 2015a). For instance, the presence of genes involved in the root hair formation indicates that the uptake of nutrients may have been transferred into glands of other vegetative organs (Ibarra-Laclette et al. 2011; Carretero-Paulet et al. 2015a). Thus, the absence of a typical root can be a result of the expression (or not expression) of specific transcription factors, and, therefore, not a result of lacking root developmental genes (Miranda, pers. comm.). In terms of a continuum or morphocline of process combinations, and in the phylogenetic context, developmental processes for ‘shoot’ (e.g. branching, cf. Fig. 9b) and developmental processes for ‘leaf’ (e.g. a determinate growth period, cf. Fig. 9b) may have been added to a ‘reduced *Pinguicula* root’. This has led in *U. dichotoma* to a runner stolon in one (shoot) pathway, and to anchor/simple stolons in another (leaf) pathway.

The PCA is a statistical method with a confidence interval for any organ and groups of organs. This leads to some standard deviation for all distances. Consequently, distances cannot be read in absolute values. Furthermore, not all modalities have the same strength in the data set, which is to some degree also dependant on the initial values defined. However, if some values are changed (e.g. from 0.5 to 1.0 or from − 1.0 to − 0.5), this does not dramatically alter the distribution of the whole data set. The modalities applied can be used for similar studies, especially on ‘unusual’ structures with mixed identities. A future investigation within the scope of dynamic morphology shall include *Pinguicula*, *Genlisea* and more *Polypompholyx* species (Reut and Płachno in prep.).

According to findings of genetic studies on *U. gibba* (Carretero-Paulet et al. 2015a), it seems that each vegetative organ of *U. dichotoma* combines developmental programs for leaf, shoot and root, which are ‘switched on’ or ‘switched off’, i.e. genes that are expressed or repressed. This way, each vegetative organ is a specific patchwork of leaf, shoot and root characters, and the developmental fate of its primordium may be triggered by chemical compounds, light, water or other environmental factors (cf. Chomicki et al. 2017; Mizutani and Kanaoka 2017; Fritz et al. 2018). Our findings in *U. dichotoma* reinforce previous observations regarding organ interchangeability and fuzzy morphology in terrestrial bladderworts (Brugger and Rutishauser 1989; Rutishauser 2016). Studies on genetic mechanisms underlying such morphogenetic patchworks have been undertaken by Katayama et al. (2010) on ‘shoot-leaf’ mixed organs of other ‘morphological misfits’, the Podostemaceae. Further genetic research on *Utricularia* is instrumental in this context (cf. Carretero-Paulet et al. 2015a; Jobson et al. 2018).

### Taxonomy

The results of the present study demonstrate that some developmental patterns of *Utricularia dichotoma* are common within the species (e.g. the initial development of a leaf and two flanking bladders at the stolon tip, i.e. in rank 1–3), and some are highly variable within and between populations (the combination of organs in rank 4 and 5, and the succession of further organs of rank 6 and higher).

The high variability of vegetative organ combinations on stolons of *U. dichotoma* mirrors the great phenotypic plasticity already known (cf. Taylor 1989; Reut and Fineran 1999, 2000). Plants from Kopouatai and Strathgordon demonstrate that population-specific patterns do exist at least with respect to primordia 4 and 5. Whether these morphological specialities are genetically determined, or directly induced by environmental conditions such as chemical compounds, hormones, light and water, needs further research on the genome and on external factors. The carve out of species, subspecies or variants from *U. dichotoma* is not well enough supported by our data due to the seemingly unlimited morphological variability of combinations of vegetative organs arising from primordia 4–10 throughout all sources studied. Furthermore, we found that the taxonomic value of anatomical characters of vegetative organs is very limited, since the anatomy of the sources investigated is largely homogenous. Therefore, our results are consistent with earlier perceptions of a highly variable *U. dichotoma* that may include many forms and previously accepted species such as *U. monanthos* and *U. novae-zelandiae*, due to similarities of vegetative and floral characters (cf. Taylor 1989; Reut and Fineran 1999) and molecular data (cf. Reut and Jobson 2010; Jobson et al. 2017) of populations from Australia (including data from Tasmania) and New Zealand (including data from Kopouatai). However, we consider that structures and patterns found in the vegetative body of *U. dichotoma* are useful in the taxonomic comparison with other species of subgenus *Polypompholyx* (Reut and Płachno in prep.).

Along with many hydrophytes, *Utricularia* has an effective vegetative reproduction, which may favour hybridization (Les and Philbrick 1993). Therefore, we cannot rule out that taxa within or closely allied with *U. dichotoma* (e.g. species or subclades of the *U. dichotoma* complex, cf. Jobson et al. 2017) are results of hybridization. Investigations on possible hybridizations are needed on several species or species groups in subgenus *Polypompholyx*, as this would add to the understanding of the morphological variability, taxonomy, distribution, evolution, and species diversity of the subgenus.

## Conclusions

*Utricularia dichotoma* exemplifies that each type of vegetative organs is a more or less stable combination of developmental processes of leaf, shoot and root characters that can develop from any defined position of the stolon node. The determination of an organ type decreases with increasing rank of a primordium in the developmental succession of vegetative organs on a stolon node. This way, the plant has flexibility in producing the appropriate organ type in fluctuating environmental conditions. However, genetically determined population-specific patterns cannot be excluded. The adaptation of *U. dichotoma* to submerged habitats is reflected by its anatomy, which shares characters with many hydrophytes. The concept of dynamic morphology and the methods we applied in our study may be a basis for future research in developmental genetics and morphology, and in the understanding of phylogenetic relationships within Lentibulariaceae and of (unusual) structures in plants in general.
